# On the Use of Chloroform in a Bite of a Rattle Snake

**Published:** 1855-09

**Authors:** 

**Affiliations:** Blue Grass, Iowa


### DR. CARPENTER ON THE USE OF CHLOROFORM IN THE BITE OF A RATTLE SNAKE.

BLUE GRASS, IOWA, June 11th, 1855.

I was called to see Miss ———, aged 13 years. Found she had been bitten by a rattle snake on the top of the right foot twenty-six hours previous to my visit. She was in severe pain with the foot and leg which was badly swollen and had assumed a very unnatural appearance, (easier imagined than described). Had not slept since the accident occurred, countenance pallid and anxious, and pulse irregular. It was plainly evident that the poison was being rapidly diffused through the system, which required immediate and energetic treatment.

℞ Brandy opt. ℥ii.

Chloroform, ℥i. M.

60 drops to be given in sweetened water every two hours.

℞ Ol Olivae,

Chloroform, *aa* ℥i. M.

Liniment to be used very freely on the foot and leg, which after a few applications, reduced the swelling and relieved the pain, while the former supported the nervous system. She recovered rapidly under the treatment. I report this case from the fact that I am not aware that Chloroform has been used in similar cases.

